# Extreme bill dimorphism leads to different but overlapping isotopic niches and similar trophic positions in sexes of the charismatic extinct huia

**DOI:** 10.1007/s00442-021-05082-8

**Published:** 2021-11-29

**Authors:** Barbara M. Tomotani, Rodrigo B. Salvador, Amandine J. M. Sabadel, Colin M. Miskelly, Julie C. S. Brown, Josette Delgado, Patrick Boussès, Yves Cherel, Susan M. Waugh, Sarah J. Bury

**Affiliations:** 1 Netherlands Institute of Ecology, NIOO-KNAW, Droevendaalsesteeg 10, 6708 PB Wageningen, The Netherlands; 2 Museum of New Zealand, Te Papa Tongarewa, Wellington, New Zealand; 3 National Institute of Water and Atmospheric Research, Wellington, New Zealand; 4 Department of Zoology, University of Otago, Dunedin, New Zealand; 5 Institut Systématique, Évolution, Biodiversité, ISYEB, Muséum national d’Histoire naturelle, Sorbonne Université, Paris, France; 6 Centre d’Etudes Biologiques de Chizé, CNRS-La Rochelle Université, Villiers-en-Bois, France; 7 Ligue pour la Protection des Oiseaux, Rochefort, France

**Keywords:** Compound-specific stable isotopes, Feeding ecology, *Heteralocha acutirostris*, Natural history collections, New Zealand, Passeriformes

## Abstract

The New Zealand huia (*Heteralocha acutirostris*) had the most extreme bill sexual dimorphism among modern birds. Given the quick extinction of the species, the cause of the dimorphism could only be hypothesised to reflect different trophic niches and reduce male/female competition. We tested that hypothesis by combining museum specimens, geometric morphometrics, and isotopic analyses. We used geometric morphometrics to describe bill shape; measured bulk (*δ*^15^N_bulk_) and (*δ*^13^C_bulk_) values from feather as proxies of the birds’ foraging habitat and diet; and compared compound-specific stable isotopes analyses (CSIA) of nitrogen in amino acids (*δ*^15^N_AA_) in male–female pairs to estimate their trophic position. Sexes had significantly different, but overlapping feather *δ*^15^N_bulk_ and *δ*^13^C_bulk_ values, but *δ*^15^N_AA_ indicated identical trophic positions and *δ*^15^N_bulk_ was not related to bill shape. Trophic position was less variable among females, consistent with a specialised foraging behaviour and, thus, supporting a partial male/female foraging segregation.

**Supplementary Information:**

The online version contains supplementary material available at 10.1007/s00442-021-05082-8.

## Introduction

**Fig. 1:**
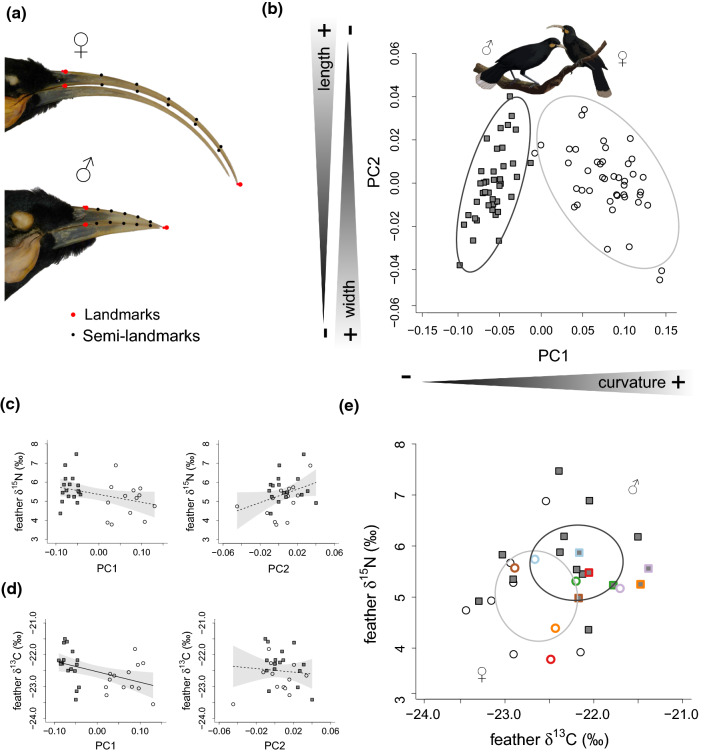
**A** Photos of male and female huia bills exemplifying the placement of landmarks. **B** PC1-PC2 biplot showing the difference in bill morphology of males and females. Each point represents one individual: white circles: females; grey squares: males. Ellipses are 95% confidence ellipses. **C** Relationship between feather *δ*^15^N_bulk_ values, PC1 and PC2. Regression lines and confidence intervals are based on model predictions. **D** Relationship between feather *δ*^13^C_bulk_ values, PC1 and PC2. **E.** Feather *δ*^15^N_bulk_–*δ*^13^C_bulk_ biplot showing the difference in isotopic niche width of males and females. Pairs of huia (males and females collected on the same date and location) are represented by the same-coloured symbols. Ellipses are 40% Standard Ellipses calculated with the R package SIBER (Jackson et al. 2011)

The extinct New Zealand huia, *Heteralocha acutirostris* (Gould 1837), was a songbird with a striking sexual dimorphism in bill shape. Males had a short and stout bill, while females had a long sickle-shaped bill (Fig. 1A). Differences were so pronounced that females and males were described (in the same publication) as different species: *Neomorpha acutirostris* Gould, 1837 and *Neomorpha crassirostris* Gould, 1837, respectively. Body size difference between the sexes of huia, however, is borderline non-significant (Moorhouse 1996).

Huia were likely distributed through the entire North Island of Aotearoa New Zealand, but declined rapidly after Polynesian settlement in the fourteenth century CE (Tennyson and Martinson 2007). They were hunted for their tail feathers, which were tapu (sacred) signifiers of rank for Māori rangatira (chiefs) and later, in the nineteenth century, were used as fashion accessories for Europeans (Potts 1884; Phillips 1963; Norman 2018). Mounted specimens in display cases were also widely traded as *objets d’art* in addition to the smaller number of specimens that ended up in museums (Tennyson and Martinson 2007; Norman 2018). In fact, by the time of European settlement, the species was already restricted to the eastern and southern portion of the North Island (Salvador et al. 2019). Its decline was officially recognised in 1890, with legal protection put in place in 1892, albeit poorly enforced (Miskelly 2014). Habitat destruction, pressure from hunting, and predation by introduced mammalian pests quickly drove the species to extinction (Tennyson and Martinson 2007). The last known specimen dates from around 1907 and is kept at the Museum of New Zealand Te Papa Tongarewa (Salvador et al. 2019).

Different hypotheses attempted to explain the huia’s puzzling bill dimorphism, especially given that the most “exaggerated” and variable trait was found in the females and not in the males. One hypothesis suggested that a pair of birds would feed cooperatively, with each sex complementing the diet of the other with the different types of prey their bills allowed them to catch (Colenso 1888; Chambers 1989; Gill and Martinson 1991). That idea was particularly prevalent in popular accounts of the huia (e.g., Phillips 1963), but it was considered to be a misinterpretation of earlier behavioural accounts (Jamieson and Spencer 1996). Other authors have proposed that the bill was a secondary sexual character used in courtship (Williams 1976), but most supported the idea that bill dimorphism was an adaptation to explore different habitat niches and avoid intra-specific competition for food (Buller 1870, 1895; Rand 1952; Selander 1966, 1972; Burton 1974; Moorhouse 1996).

Such a niche separation hypothesis relies on morphoanatomical and ecological data, which is difficult to support with unambiguous evidence, especially for vertebrates (Darwin 1874; Hedrick and Temeles 1989; Shine 1989; Fairbairn et al. 2007). The extinction of this species happened so fast that there was little time to gather natural history and ecological data. As early as 1870, Buller recognised the species would soon be extinct and naturalists should record all they could. However, most contemporaneous accounts relate to collection trips and restrict their accounts to how many birds were shot each day (e.g. Dieffenbach 1843; Buller 1888; Medway 1968). Thus, data on huia dietary items and feeding comprise anecdotal behavioural observations or limited studies in captivity (Buller 1870, 1888; Potts 1884). Fortunately, the collection frenzy has left us with a reasonable assortment of huia specimens in natural history collections worldwide (Salvador et al. 2019).

Natural history collections have been receiving renewed interest as an increasing number of researchers are starting to realise their potential for ecological studies (Meineke et al. 2019; Jonathan et al. 2019; Salvador and Cunha 2020). Museum specimens represent long-term time series with broad geographic coverage and, locked away in each specimen, there is the possibility of exploring data relating to morphometrics, genetics, and stable isotopes (Chamberlain et al. 2005; Webster 2018; Meineke et al. 2019). Whilst the broader questions investigated using such collections usually pertain to anthropogenic changes, macroevolution and species decline, the possibilities go beyond that: current techniques, when applied to preserved specimens, can reveal additional aspects of ecology and behaviour, enabling a more comprehensive reconstruction of the biology of extinct species.

Determining the trophic niche of species, in particular omnivores and higher order carnivores, can be challenging if feeding history cannot be thoroughly assessed, as is the case in the huia. However, it is possible to gain insight into foraging and diet using bulk stable isotope ratios of nitrogen (^15^N/^14^N) and carbon (^13^C/^12^C) (*δ*^15^N_bulk_ and *δ*^13^C_bulk_, respectively), which can be measured in feathers of museum specimens (Hobson and Clark 1992a, b). Values of *δ*^15^N_bulk_ have traditionally been used to estimate trophic position (DeNiro and Epstein 1981; Minagawa and Wada 1984; Peterson and Fry 1987; Post 2002), whilst *δ*^13^C_bulk_ data are more useful for determining sources of carbon for an organism (Rounick and Winterbourn 1986; Peterson and Fry 1987; O’Leary et al. 1992; Post 2002). With the introduction of compound-specific stable isotope analysis (CSIA) of nitrogen in amino acids, it is now possible to more accurately assess an animal’s trophic position (TP) using *δ*^15^N values of certain amino acids (*δ*^15^N_AA_) (Chikaraishi et al. 2011, 2014; Whiteman et al. 2019, 2021). This approach is based on the fractionation between the so-called ‘trophic’ and ‘source’ amino acids in metabolic processes. Whilst the former results in an enrichment in ^15^N as biomass is transferred from one trophic level to another, thereby increasing *δ*^15^N values in secondary consumers, the latter shows negligible enrichment (Chikaraishi et al. 2014; McMahon and McCarthy 2016; Cherel et al. 2019). Thus, a general equation based on *δ*^15^N_Glx_ (glutamic acid, trophic) and *δ*^15^N_Phe_ (phenylalanine, source) can be used to assess the TP of any organism across different terrestrial environments (Chikaraishi et al. 2009, 2011, 2014).

Herein, we used *δ*^15^N_bulk_, *δ*^13^C_bulk_ and *δ*^15^N_AA_ analyses combined with bill geometric morphometrics to elucidate the enigmatic huia bill shape sexual dimorphism and its relationship to differences in feeding behaviour and ecology. We specifically tested whether males and females occupied distinct trophic niches, which form the basis of the competition hypothesis (Buller 1870, 1895; Rand 1952; Selander 1966, 1972; Burton 1974; Moorhouse 1996).

## Materials and methods

### Geometric morphometrics

To describe male and female morphological differences in bill shape, we employed geometric morphometrics analysis using quality photos of huia bills from skins and mounts from ten natural history collections (84 adult specimens: 43 ♀♀, 41 ♂♂, collected from 1868 to 1907; see Supplementary Material 2). From approximately 100 natural history collections that we contacted worldwide, only 31 had specimens of huia. The full list of specimens we could locate has been published in Salvador et al. (2019).

The specimens were positioned on right lateral view, making sure the bill was parallel to the plane of the photograph, with a measurement scale placed at the same level as the bill. Three landmarks (henceforth LM or, plural, LMs) were plotted on the photographs on the upper maxilla, which included: upper distal limit of nare (LM1); tip of the upper maxilla (LM2); lower distal limit of nare (LM3). Using the software MakeFan 8 (Integrated Morphometrics Package Suite 8; Sheets 2014), we fitted five equidistant semi-landmarks between LM1 and LM2, and five between LM2 and LM3. This totalled 13 LMs for the analysis. The photos were set to scale and the coordinates were acquired using the software ImageJ v.1.52a (Schneider et al. 2012).

Coordinates were then standardised through Procrustes fitting (Rohlf and Slice 1990; Dryden and Mardia 1998) using the software PAST v.3.25 (Hammer et al. 2001). Procrustes standardises positioning and scale/size, so that the analysis can focus solely on the shape (Rohlf and Slice 1990; Dryden and Mardia 1998). A principal component analysis (PCA) was conducted using the standardised coordinates in PAST. The graphical interface of that software was also used to visualise the influence of the relative warps (principal components) on bill shape via vectors and thin-plate splines (Dryden and Mardia 1998).

### Feather sampling

We collected feather samples from skins and mounts belonging to eight of the ten natural history collections mentioned above, so all samples came from specimens also used for morphometric measurements. We focused on adult birds with provenance data and the year they were collected, taking (randomly) one contour feather from the breast area. We managed to gather feathers from 30 specimens (13 ♀♀, 17 ♂♂; see Supplementary Material 2) for bulk isotope analysis.

For *δ*^15^N_AA_ analysis, our aim was to minimise isotopic differences due to temporal and environmental variables. We thus selected individuals from the 30 specimens above, by matching them into male–female “pairs” based on the same collection date and location. This greatly increased the likelihood of obtaining true mating pairs of birds, since it was typical for past collectors to shoot a male–female pair of birds together. Given that huia were monogamous, highly territorial and non-migratory (Moorhouse 1996), this choice also ensures that feather stable isotope values can be compared with confidence.

From all available specimens of huia (Salvador et al. 2019), we identified nine pairs suitable for *δ*^15^N_AA_ analysis. We could only obtain samples of 6 of those pairs (12 birds; see Supplementary Material 2), which were part of the samples from the bulk isotope analysis above.

All feathers were cleaned in ethanol prior to subsampling for stable isotope analyses. The top 1.5 cm of each feather was cut off using scissors cleaned in ethanol, then the two vanes of the section were separated from the shaft and used for analysis; the shaft was discarded. For each feather sample, the vanes were homogenised by snipping them into fine segments. Approximately, 0.5–0.8 mg of the homogenised feather sub-sample was then weighed for bulk stable isotope analysis and around 1.0 mg was weighed for CSIA.

### Bulk stable isotope analysis

Bulk stable isotope analysis was carried out on a DELTA V Plus continuous flow isotope ratio mass spectrometer (IRMS) linked to a Flash 2000 elemental analyser using a MAS 200 R autosampler (Thermo-Fisher Scientific, Bremen, Germany) at the NIWA Environmental & Ecological Stable Isotope Facility (Wellington, New Zealand). ISODAT (Thermo-Fischer Scientific) software calculated *δ*^15^N values against an atmospheric air international standard. Values of *δ*^13^C_bulk_ were calibrated against a CO_2_ reference gas, relative to the international standard Carrara Marble NSB-19 (National Institute of Standards and Technology, NIST; Gaithersberg, MD, USA), which in turn, was calibrated against the original Pee Dee Belemnite (PDB) limestone standard and was then corrected for ^17^O. Stable isotope ratios were expressed as delta values (*δ*) in per mil units (‰), which represent the ratios of heavy to light isotopes within a sample (*R*_sample_), relative to the ratio in an international standard (*R*_standard_) as:

1 $$ \delta = \left( {\left( {\frac{{R_{{\text{e}}} }}{{R_{{{\text{standard}}}} }}} \right){-} 1 } \right) \times 1000. $$

Reference materials were used to determine isotopic values following Paul et al. (2007). Sample *δ*^15^N values were two-point normalised using isotopic data from the daily analysis of National Institute of Standards and Technology NIST 8573 United States Geological Society USGS40 L-glutamic acid and NIST 8548 Institute of Atomic Environmental Agency IAEA-N-2 ammonium sulphate. Sample *δ*^13^C values were two-point normalised using isotopic data from the daily analysis of NIST 8573 USGS40 L-glutamic acid and NIST 8542 IAEA-CH-6 Sucrose. Precision was determined by the repeat analysis of working laboratory standards dl-Leucine (dl-2-amino-4-methylpentanoic acid, C_6_H_13_NO_2_, Lot 127H1084, Sigma, Australia), L Proline and “NIWA squid”. Precision for DL Leucine, for which we had the greatest replication, was 0.14‰ (± 1 standard deviation, S.D., *n* = 322) for *δ*^13^C_bulk_, and 0.17‰ (± 1 S.D., *n* = 316) for *δ*^15^N_bulk_. Data from the analysis of the international standard USGS65 glycine (*n* = 45) were used to check accuracy and precision. Values were accurate to within 0.08‰ for *δ*^13^C_bulk_ and to within 0.14‰ for *δ*^15^N_bulk._

Due to a global increase in atmospheric carbon levels caused by anthropogenic activity (Suess effect), we used formula #4 of Verburg (2007) to correct *δ*^13^C_bulk_ measurements, considering the year each specimen was collected and starting at 1840 (differences between the corrected and uncorrected bulk carbon isotope varying from 0.0002 to 0.02). In general, the correction factors were low, with almost all values within the accuracy of *δ*^13^C_bulk_ measurements (0.14‰).

### Amino acid hydrolysis, derivatisation, and compound-specific stable isotope analysis of nitrogen in amino acids

Feathers were first hydrolysed into individual amino acids with 6 N HCl, then esterified using acetyl chloride-isopropanol followed by trifluoroacetylation using trifluoroacetic anhydride in dichloromethane. The method is fully documented in Hannides et al. (2009), which draws on earlier reported methods (Ueda et al. 1989; Silfer et al. 1991; Metges et al. 1996; Macko et al. 1997; Veuger et al. 2005; Popp et al. 2007). There were a few minor variations from Hannides et al. (2009) which are detailed in Supplementary Material 1. An external standard amino acid mix and an in-house quality control sample “NIWA Squid” were derivatised at the same time as the samples following the same protocol. The amino acid mix external standard comprised a suite of six commercially available amino acid reference materials: glycine (Gly), threonine (Thr), leucine (Leu), proline (Pro), glutamic acid (Glx) and phenylalanine (Phe) (see Supplementary Material 1 for further information). During the hydrolysis step, asparagine is converted to aspartic acid (Asx) and glutamate is converted to glutamic acid (Glx). Derivatised samples were taken up in ethyl acetate before analysis on the gas chromatography-isotopic ratio mass spectrometer (GC-IRMS). Eleven amino acids were detected and reported, which fell into the following groups: seven trophic amino acids: alanine (Ala), Asx, Glx, isoleucine (Ile), Leu, Pro, valine (Val); two intermediate amino acids: Gly, serine (Ser) (as defined by Shen et al. 2021); a source amino acid: Phe; and a metabolic amino acid: Thr. See also Cherel et al. (2019: sect. 2.3) for further information on these amino acids.

CSIA was carried out on a TRACE Ultra Gas Chromatograph with GC IsoLink interface via a ConFlo IV interface to a DELTA V Plus IRMS (Thermo Fisher Scientific, Bremen, Germany), with GC PAL autosampler (CTC Analytics, Switzerland). Derivatised amino acids were separated on an Agilent J&W DB-5 ms column (60 m × 0.25 mm ID × 0.25 µm film thickness), then combusted/reduced at 1000 °C in the GC Isolink furnace. The carbon dioxide from the combustion was removed with a liquid nitrogen trap prior to sample introduction into the IRMS via the Conflo IV open split.

Triplicate measurements of each sample were bracketed by the amino acid external standard referred above. The *δ*^15^N value of each amino acid in the external standard was calibrated against international standards (e.g., USGS-40, USGS-41) using the EA-IRMS system, enabling the correction of sample *δ*^15^N_AA_ values. Finally, the in-house quality control sample “NIWA squid” was used to monitor the reproducibility of the hydrolysis, derivatisation process and IRMS analysis across the batches. Precision on replicates for *δ*^15^N_AA_ values was always better than ± 1‰.

### Data analysis

All statistical analyses were carried out in R 4.0.2 (R Core Team 2020). When using models, model selection was carried out via backwards selection, dropping non-significant terms at each step. See Supplementary Material 3 for the R code.

Male and female morphological differences: To investigate the bill shape differences between males and females, we used the function “procD.lm” from the R package “geomorph” (Adams and Otárola‐Castillo 2013) to perform a Procrustes ANOVA (randomised residuals, 1000 iterations) with sex as an explanatory variable. We also used linear models to test whether the separated PC1 and PC2, which explained the different aspects of bill shape were related to sex.

Bulk stable isotopes and morphology: We tested the relationship between *δ*^15^N_bulk_ and *δ*^13^C_bulk_, and bill morphology. For that, we used multiple regression analyses, including PC1, PC2 and the interaction between PC1 and PC2 as explanatory variables to test if the variation in *δ*^13^C_bulk_ and *δ*^15^N_bulk_ values was correlated with the variation in bill morphology.

Male and female bulk stable isotope differences: To test if sexes differed in diet and foraging, we first looked at male/female differences in *δ*^15^N_bulk_ and *δ*^13^C_bulk_ in their feathers. We used linear models testing *δ*^15^N_bulk_ and *δ*^13^C_bulk_ separately and then carried out a multivariate analysis of variance (MANOVA) with both *δ*^15^N_bulk_ and *δ*^13^C_bulk_ as response variables. We also used the package SIBER (Jackson et al. 2011) to assess whether the width of the male and female isotopic niches differed via a comparison of the posterior distribution of the sexes’ Standard Ellipse Areas (SEAc, iterations = 10^5^, burning = 10^3^, thin = 10, chain = 2). The ellipses were also used to calculate the amount of niche overlap between males and females. Finally, we compared whether pairs of birds (males and females with the same collection location and date) were consistently different, in other words, if within a pair, the males would have consistently higher or lower values of *δ*^15^N_bulk_ and *δ*^13^C_bulk_ than females. We first used a Wilcoxon signed-rank test to look independently at *δ*^15^N_bulk_ and *δ*^13^C_bulk_ values. Then, we took a multivariate approach to compare both *δ*^15^N_bulk_ and *δ*^13^C_bulk_ values together and used a Hotelling’s T2 test.

Male and female δ^15^N_AA_ values and trophic position differences: While a relative TP (assessed simply from the difference between *δ*^15^N_Glx_ and *δ*^15^N_Phe_ values) allows for comparison between individuals, the lack of studies on land birds precludes its full use for TP assessment. Hence, we used *δ*^15^N_Glx_ and *δ*^15^N_Phe_ values to estimate the TP of each individual of the pair using the formula described in Chikaraishi et al. (2014) for terrestrial systems:

2 $$ {\text{TP}} = \frac{{\delta^{15} {\text{N}}_{{{\text{Glx}}}} - \delta^{15} {\text{N}}_{{{\text{Phe}}}} + 8.4}}{7.6} + 1, $$

where *β* represents the isotopic difference between the trophic AA, *δ*^15^N_Glx_, and the source AA, *δ*^15^N_Phe_, in terrestrial primary producers, which in New Zealand are almost exclusively C3 plants. A *β* value of 8.4‰ is used, which is the conventional average value from Chikaraishi et al. (2009, 2011). Beta values in plants can vary according to photosynthetic pathway, habitat, vascularization, tissue type, and taxonomic group (Kendall et al. 2019; Ramirez et al. 2021). However, given the many unknowns regarding the huia and its habitat, we consider that the conventional 8.4‰ is the most acceptable compromise. The trophic enrichment factors (TEF) represent the isotopic enrichment of both *δ*^15^N_Glx_ and *δ*^15^N_Phe_ values with each trophic step which is estimated to be 7.6‰. We also took the opportunity to verify whether the above parameters (β and TEF) match expectations against what we know about the huia’s feeding behavior.

As before, we first tested if males and females differed on average in their TP using linear models and then used the Wilcoxon signed-rank test for a paired analysis. We also tested if individual *δ*^15^N_AA_ values differed between males and females with linear models, with a Holm–Bonferroni correction on *p* values to account for multiple testing.

When models are used, statistics were extracted at the point of the exclusion of the term from the model. Values for model estimates and raw values are given as means ± SE.

## Results

### Male and female morphological differences

PC1 explained 92.63% of the variation in bill shape; it is related to the curvature of the bill and hence, it represents the overall bill shape. PC2 explained 4.49% of the variation, being related to both bill height and length. The effect of variations in the PCs was visually assessed using thin-plate splines (Supplementary Material 1: Fig. S2).

There was a clear distinction between males and females in bill shape (SS = 0.40, MS = 0.40, *Z* = 5.82, *p* < 0.01), with sexes also significantly differing in their PC1 (*F*_1,82_ = 507.73, *p* < 0.01) with no overlap (females: 0.07 ± 0.004, males: − 0.07 ± 0.004), but not in PC2 (*F*_1,82_ = 0.09, *p* = 0.76). Thus, a pronounced bill curvature is only found in females, while females and males present a similar degree of morphological variation in bill height and length (Fig. 1B).

### Bulk stable isotopes and morphology

There was no relationship between *δ*^15^N_bulk_ and PC1 (*F*_1,28_ = 3.77, *p* = 0.06) or with PC2 (*F*_1,28_ = 3.86, *p* = 0.06). There was a significant relationship between *δ*^13^C_bulk_ and PC1 (*F*_1,28_ = 6.69, *p* = 0.02, slope = –3.31 ± 1.28), but no relationship with PC2 (*F*_1,28_ = 0.04, *p* = 0.83). In neither case was there a significant effect of the interaction between PC1 and PC2. Thus, there is a correlation between bill height and length (PC2) and *δ*^13^C_bulk_ but no relationship between the variation in bill shape (PC1) and the variation in *δ*^15^N_bulk_ and *δ*^13^C_bulk_ values (Fig. 1C, D).

### Male and female bulk stable isotope differences

Males had, on average, significantly higher *δ*^15^N_bulk_ values (females: 5.02 ± 0.22‰, males: 5.67 ± 0.20‰) and *δ*^13^C_bulk_ values (females: -22.77 ± 0.14‰, males: -22.31 ± 0.12‰) regardless of whether the isotopes were tested independently (*δ*^15^N_bulk_: *F*_2,28_ = 4.85, *p* = 0.04, *δ*^13^C_bulk_: *F*_2,28_ = 6.00, *p* = 0.02) or together in the same analysis (Pillai = 0.28, *F*_2,27_ = 5.27, *p* = 0.01). This suggests that males and females occupied distinct, but overlapping isotopic niches (Fig. 1E). The isotopic niche width, however, was not different between males and females (males SEAc = 1.30‰^2^; females SEAc = 1.41‰^2^), since the probability of obtaining a smaller ellipse for males was 57%. The amount of overlap of the 40% Standard Ellipses was 28% relative to the female’s ellipse and 31% relative to the male’s (Fig. 1E).

When *δ*^15^N_bulk_ and *δ*^13^C_bulk_ were investigated in pairs of birds, there was a significant difference in *δ*^13^C_bulk_ values (*V* = 21, *p* = 0.03; female subset: − 22.51 ± 0.17, male subset: -21.96 ± 0.14), but not in *δ*^15^N_bulk_ values (*V* = 16, *p* = 0.31; female subset: 4.99 ± 0.31, male subset: 5.40 ± 0.13). In the multivariate analysis, males within a pair had significantly higher *δ*^15^N_bulk_ and *δ*^13^C_bulk_ values (T2 = 20,413.23, *df*1 = 2, *df*2 = 4, *p* < 0.01). Thus, the average difference in isotopes between males and females was also present in the subset of males and females used for comparing pairs of birds (Fig. 1E).

### Male and female *δ*^15^N_AA_ values and trophic position differences

**Fig. 2:**
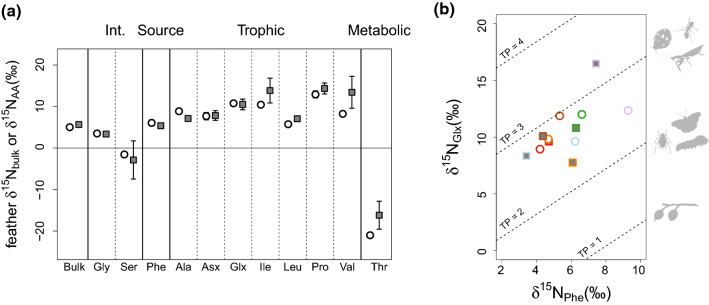
**A** Feather *δ*^15^N_bulk_ and *δ*^15^N_AA_ values for each of the analysed amino acids: trophic: aspartic acid (Asx), alanine (Ala), glutamic acid (Glx), isoleucine (Ile), leucine (Leu), proline (Pro), valine (Val); intermediate (Int.): glycine (Gly), serine (Ser); source: phenylalanine (Phe); metabolic: threonine (Thr). White circles: females; grey squares: males. **B** Cross plot of the feather *δ*^15^N_Glx_ and *δ*^15^N_Phe_ values for all samples of huia analysed (circles males, squares females); different colours represent distinct pairs (as in Fig. 1). The TP of distinct invertebrates are based on Chikaraishi et al. (2014): TP = 1, fruits, berries, seeds, nectar; TP = 2, aphid, adult and larval Lepidoptera; TP = 3, coccinellid, mantid, ant

There were no differences in TP between males (2.77 ± 0.13) and females (2.73 ± 0.07), either when analysing the average differences (*F*_1,10_ = 0.10, *p* = 0.75), or in a paired test (V = 10, *p* = 1.00). There was also no difference in any *δ*^15^N_AA_ values (Fig. 2A; Gly: *F*_1,6_ = 0.03, *p* = 0.87; Ser: *F*_1,7_ = 0.06, *p* = 0.81; Phe: *F*_1,10_ = 0.49, *p* = 0.49; Ala: *F*_1,2_ = 7.94, *p* = 0.11; Asx: *F*_1,10_ = 0.01, *p* = 0.92; Glx: *F*_1,10_ = 0.03, *p* = 0.86; Ile: *F*_1,7_ = 0.99, *p* = 0.35; Leu: *F*_1,8_ = 2.65, *p* = 0.14; Pro: *F*_1,10_ = 0.82, *p* = 0.38;Val: *F*_1,7_ = 1.41, *p* = 0.27; Thr *F*_1,7_ = 1.59, *p* = 0.24). Females displayed a smaller variation in *δ*^15^N_AA_ values (Fig. 2A) to males and in TP (Fig. 2B; females’ range: 2.51–2.97, males’ range 2.33–3.30).

## Discussion

In this study, we illustrated how museum specimens shed light into the puzzling bill sexual dimorphism of the huia, a charismatic extinct endemic bird from New Zealand. Despite having striking sexual difference in bill shape, males and females did not show large differences in their isotopic niche and trophic position as would be expected. Instead, they had different, but overlapping, feather *δ*^15^N_bulk_ and *δ*^13^C_bulk_ values and identical trophic positions, with a smaller variation in females. Thus, our results point to a partial sexual trophic segregation, with a higher specialisation in females.

Our results concur with previous studies (e.g. Buller 1888; Jamieson and Spencer 1996) showing large differences in bill shape in males and females huia, with virtually no intermediate phenotypes. However, this difference is driven by bill curvature (captured by PC1, which explains over 90% of the variation) rather than bill length or height. Despite these strong morphological differences, we found no evidence for a relationship between bill shape and *δ*^15^N_bulk_ and *δ*^13^C_bulk_ values, which could be expected if birds with distinct bill shapes were feeding on largely isotopically distinct food items.

Moreover, sexes had different, but overlapping, feather *δ*^15^N_bulk_ and *δ*^13^C_bulk_ values, with males having higher *δ*^15^N_bulk_ values. That suggests that males would occupy a higher TP than females, but *δ*^15^N_AA_ values did not fully support that scenario. Instead, this apparent higher TP of males seems to be caused by a larger between-individual variation of *δ*^15^N_AA_ values in males than in females (Fig. 2A). The narrower variation in female TP, however, would be consistent with a more specialised foraging behaviour, suggesting that males and females likely employed distinct foraging strategies. For both sexes, TP values (~ 2.7) indicated that the diet of huia birds was in all likelihood composed mainly of primary consumers, with occasional vegetable components (Fig. 2B).

Indeed, most available literature solely refers to the larvae of the huhu beetle (*Prionoplus reticularis* White, 1843) as a food source, supposedly based on direct observation (Buller 1870; Potts 1884). Buller (1888) also mentioned tree weta (*Hemideina thoracica* (White, 1846), Orthoptera), but did not indicate if both sexes of the huia used this food source. The same author also observed huia likely feeding on the ground and noted other unidentified insects as a food source, as well as berries from hinau (*Elaeocarpus dentatus*), pokaka (*Elaeocarpus hookerianus*), pigeonwood (*Hedycarya arborea*), and karamu (*Coprosma robusta*), with the possibility of titoki berries (*Alectryon excelsum*) and the fleshy cones of kahikatea (*Dacrycarpus dacrydioides*). Other prey items have been reported from stomach content analyses, but without mention of the sex of the bird or life stage of the prey. These include: Lepidoptera larvae; Coleoptera larvae and adults; Diptera (uncertain if larvae or adults); Orthoptera (likely referring to weta); New Zealand mantis (*Orthodera novaezealandiae* (Colenso, 1882)); spiders; hinau, pigeonwood, tōwai (*Weinmannia silvicola*), kaikōmako (*Pennantia corymbosa*), and other undetermined berries or seeds (Dieffenbach 1843; Buller 1870, 1888, 1892). The larger variation in TP of male huia in our data set suggests that males could access a broader selection of food sources than females (Fig. 2B). Overall, the TP estimated with Eq. (2) yielded coherent numbers with regards to what has been observed of the huia’s feeding behaviour. This reinforces the appropriateness of both parameters β and TEF in the equation for estimating TP of terrestrial birds.

Huia were historically distributed in a restricted area of New Zealand’s North Island (Salvador et al. 2019) when the specimens were collected; our samples were likewise restricted to a few decades in temporal ‘range’ until the species became extinct (1868–1907). Moreover, the huia was a monogamous and highly territorial species (Moorhouse 1996) and birds were regularly found either in pairs or family groups. Thus, it is unlikely that the differences in *δ*^15^N_bulk_ and *δ*^13^C_bulk_ values that we found are caused by artefacts such as location, collection date (seasonal) differences, or habitat degradation; all of which would impact *δ*^15^N and *δ*^13^C values (Chikaraishi et al. 2011).

A few anecdotal accounts have suggested that the huia bill differences could be related to different foraging strategies. Buller (1870) kept a pair of birds in captivity for over a year and described that, when presented with a decaying log full of larvae (most likely Coleoptera), the male would prod the more decayed parts, chiselling and moving chunks of wood out of the way to reach prey (Buller 1888; Burton 1974), while the female used her long and pliable bill to probe the harder parts that the males could not reach (Buller 1870, 1873). In the field, both males and females were typically seen on high branches of trees searching for insects, and both sexes were observed removing mosses and ferns from tree branches to get to the bark and thus, their prey (Potts 1884). Males were seen on high branches tearing off pieces of bark (Buller 1870), but it is not clear whether females could also perform this activity (Potts 1884).

Different hypotheses have been proposed for the sexual dimorphism in huia and other bird species, with the sex competition hypothesis being the most accepted. This hypothesis states that a niche segregation would allow sexes to avoid competition within the pair and thus feed in close proximity. Selander (1966) argued that the possibility of expanding feeding niches between sexes is associated with reduced competition from other species with similar foraging ecology typical of island environments. This idea is supported by a lower degree of dimorphism in tremblers (Passeriformes: Mimidae) on islands where competitors are present (Storer 1989) and it closely matches the huia’s case, where no direct competitor was available. The endemic North Island saddlebacks, *Philesturnus rufusater* (Lesson, 1828), forage under bark (in a similar way to male huia, albeit lacking specialised musculature; Burton 1974) and leaf litter for invertebrates, but saddlebacks are much smaller and are not particularly selective about their foraging habits (Atkinson and Campbell 1966). Another possible competitor that chisels wood in search of larvae, the endemic kaka *Nestor meridionalis* (Gmelin, 1788), is a much larger bird and is not strictly selective (Beggs and Wilson 1987). That leaves the female huia as the only specialist wood-prober among New Zealand endemic birds (Moorhouse 1996). Given the bill morphology of the huia’s most closely related genus in this regard, the saddlebacks (*Philesturnus* spp.; Shepherd and Lambert 2007), it is much more likely that males retained the ancestral form, whilst females were selected for a derived bill shape.

Although the huia is an extreme example, other bird species present varying degrees of bill dimorphism that represent cases of niche segregation (Jamieson and Spencer 1996). The green wood hoopoe, *Phoeniculus purpureus* (J.F. Miller, 1784), exhibits opposite sex-based bill traits to the huia (although on a smaller scale): females have chiselling bills and males have long curved bills, feeding on distinct invertebrate prey at different levels of the canopy (Radford and Plessis 2003, 2004). One interesting possibility is that the differences in *δ*^13^C_bulk_ in huia, higher on average in males, could indicate that females and males had a preference for foraging at different levels of the forest, similar to what is seen in green wood hoopoes (Radford and Plessis 2003), because tree food sources on the top of the canopy tend to be ^13^C-enriched relative to the ground sources (France 1996). It is difficult to be certain of that, though, given that Potts (1884) observed both male and females foraging on the higher branches of trees; however, we cannot discard the possibility that males would spend more time in the canopy than females.

Bill dimorphism and niche segregation has also been observed in other birds, such as woodpeckers, Hawaiian honeycreepers, sunbirds, hummingbirds, and tremblers, among others (Selander 1966; Storer 1989; Temeles et al. 2000, 2010). The niche segregation hypothesis suggests that if no niche segregation exists, then *δ*^15^N and *δ*^13^C values would be the same between the two sexes; however, niche segregation can still occur when there is no difference between carbon and nitrogen isotope values (e.g., Navarro et al. 2009). We found different, but overlapping, feather *δ*^15^N_bulk_ and *δ*^13^C_bulk_ values, though *δ*^15^N_AA_ values indicated identical TP. However, TP was less variable among females, which is consistent with a more specialised foraging behaviour (i.e., males had a more varied diet). Thus, our results support a partial male/female foraging segregation, which could have enabled huia to avoid sexual competition for food items in their territory.

Pacific rats (kiore), *Rattus exulans* (Peale, 1848), were introduced in New Zealand in the late thirteenth century CE (Wilmhurst et al. 2008), but the extent of their impact on the huia is unknown. However, sub-fossil material indicates that huia were more broadly distributed through the North Island of New Zealand prior to Polynesian settlement (Tennyson and Martinson 2007; Salvador et al. 2019). It is possible that rats might have competed for part of the birds' resources (i.e., large invertebrates), which could consequently have narrowed the huia's niche and made the diets of male and female huia more similar by the time of European settlement and specimen collection. This hypothesis could potentially be tested by comparing isotopic signatures from sexed huia bones sourced from pre-human bone deposits.

## Conclusion

The unique features of the huia make them a good model to tackle questions of sexual segregation in diet. It was after all, the avian example chosen by Darwin (1874) to exemplify natural selection-driven sexual dimorphism as opposed to sexual selection. However, the rapid extinction of huia hampered any effort to collect accurate behavioural information. Here, we used museum specimens to address this question and show that it is possible to elucidate aspects of behaviour and ecology even in extinct and poorly known species. This was only possible thanks to the abundance of curated bird specimens in museums worldwide.

When museum specimens are used in a combination with various analytical approaches and lines of evidence, a wealth of ecological information that is not otherwise available can be gathered. Museum specimens are only recently being recognised as important repositories of samples and information that can go beyond systematics and/or population genetics, in answering broader ecological and evolutionary questions, in addition to reconstructing the behaviour of extinct species (Lambert et al. 2009; Salvador and Cunha 2020). Thus, our study highlights the importance of the continued effort in the collection and curation of specimens in natural history collections.

## Supplementary Information

Below is the link to the electronic supplementary material.

Supplementary file1 (PDF 730 kb)

Supplementary file2 (CSV 50 kb)

Supplementary file3 (R 29 kb)

## Data Availability

All data can be found in the text and Supplementary Material.
