# Supplementary material for: Extreme bill dimorphism leads to different but overlapping isotopic niches and similar trophic positions in sexes of the charismatic extinct huia
Source: Oecologia. 2021 Nov 29;198(1):67–77. doi: 10.1007/s00442-021-05082-8 (PMC8803797; doi:10.1007/s00442-021-05082-8)
Supplement: Supplementary file 1 — Supplementary file1 (PDF 730 kb) [file 442_2021_5082_MOESM1_ESM.pdf]

Extreme bill dimorphism leads to different but overlapping isotopic niches and similar trophic positions in sexes of the charismatic extinct huia

Barbara M. Tomotani[1,2], Rodrigo B. Salvador[2], Amandine J. M. Sabadel[3,4], Colin M. Miskelly[2], Julie C. S. Brown[3], Josette Delgado[3], Patrick Bousses[5], Yves Cherel[6], Susan M. Waugh[7], Sarah J. Bury[3]

[1] Netherlands Institute of Ecology, NIOO-KNAW. Wageningen, The Netherlands.
[2] Museum of New Zealand Te Papa Tongarewa. Wellington, New Zealand.
[3] National Institute of Water and Atmospheric Research. Wellington, New Zealand.
[4] Department of Zoology, University of Otago, Dunedin, New Zealand
[5] Institut Systématique, Évolution, Biodiversité, ISYEB, Muséum national d'Histoire naturelle, Sorbonne Université. Paris, France.
[6] Centre d'Etudes Biologiques de Chizé, CNRS-La Rochelle Université. Villiers-en-Bois, France.
[7] Ligue pour la Protection des Oiseaux. Rochefort, France.

Methods

Wet chemistry method for chemical extraction, purification, and derivatisation of samples for compound-specific stable isotope analysis (CSIA) of nitrogen in amino acids (N-AA) (after Hannides et al. 2009)

The Hannides et al. (2009) protocol is reproduced below, with the variations applied by the NIWA laboratory indicated in italics (sometimes in brackets):

1 mL (*0.5 mL*) of 6N HCl were added to 5 mg (10-20 mg) of homogenised sample in each reaction vial. Vials were flushed with N_2 gas and hydrolysed on a heating block at 150°C for 70 minutes, then evaporated to dryness at 55°C under a stream of N_2 (~1-2 hours). They were redissolved in 1 mL 0.01N HCl then filtered with low-protein binding filters to remove particles. The hydrolysate was further purified using cation-exchange chromatography (Metges et al. 1996) with a 5-cm column of resin prepared in a glass Pasteur pipette. AAs on the column were eluted with repeated rinses of 2N NH_4OH, and the eluant was evaporated to dryness under a stream of N_2 at 80ºC. Finally, the samples were reacidified with 2 mL (*5 mL*) 0.2N HCl, heated at 110ºC for 5 min and evaporated to dryness under a stream of N_2 at 110ºC (*55ºC*). Hydrolysed samples were esterified with 2.0 mL (*2.5 mL*) of 4:1 isopropanol and acetyl chloride mixture, heated to 110ºC for 60 min following a N_2 flush. The esterified samples were dried under a stream of N_2 at 60ºC, and 1 mL (*800 µL*) of 3:1 methylene chloride:trifluoracetic anhydride (TFAA) was added. Samples were acylated by heating to 100ºC for 15 min after a N_2 flush (trifluoroacetylation step). The derivatized samples were further subject to purification by solvent extraction following Ueda et al. (1989).The TFA derivatives were evaporated at room temperature, *under a stream of N_2* and redissolved in 3 mL 1:2 chloroform:P-buffer (KH_2PO_4 + Na_2HPO_4 in Milli-Q water, potential Hydrogen [pH] 7), *placing the vials in a vortex for 60 secs*. This transfers the amino acids to the chloroform fraction with contamination going into the P-buffer.

After sonication and centrifugation (10 min at 600 g), the chloroform fraction containing solely the acylated AA esters was removed and the solvent extraction process repeated. Finally, to ensure complete derivatization, the chloroform was evaporated at room temperature *under a stream of N_2*, and the acylation step was repeated. All samples were stored in 1 mL (*800 µL*) of 3:1 methylene chloride:TFAA at 4ºC and analysed immediately where possible. If analysed immediately samples were dried under a stream of N_2 and taken up in ethyl acetate and diluted to the appropriate concentration for analysis on the gas chromatography-isotopic ratio mass spectrometer. With this above procedure, nine samples (*up to 27 samples/standards*) can be prepared for AA analysis in two (*three*) days. *If it was not possible to analyse samples immediately after derivatisation, samples were re-derivatised just before analysis by drying the sample down under N_2, adding 0.5 mL of TFAA and 0.5 mL of ethyl acetate, leaving to stand at room temperature for an hour, then evaporating to dryness at room temperature. The sample was then diluted with about 100 µL of ethyl acetate and run on the CSIA IRMS system immediately (within a few hours).*

Correction of raw δ^{15}N data

Correction of raw δ^{15}N data was carried out by plotting the mean δ^{15}N value of each individual amino acid standard measured on the GC-Isolink IRMS system versus. their ‘true’ value measured on an EA-IRMS (Fig. S1; EA-IRMS measurements carried as described in the Methods section). Our R2 is always > 0.98. Raw δ15N values for amino acids in our samples were then corrected using the fitting equation.

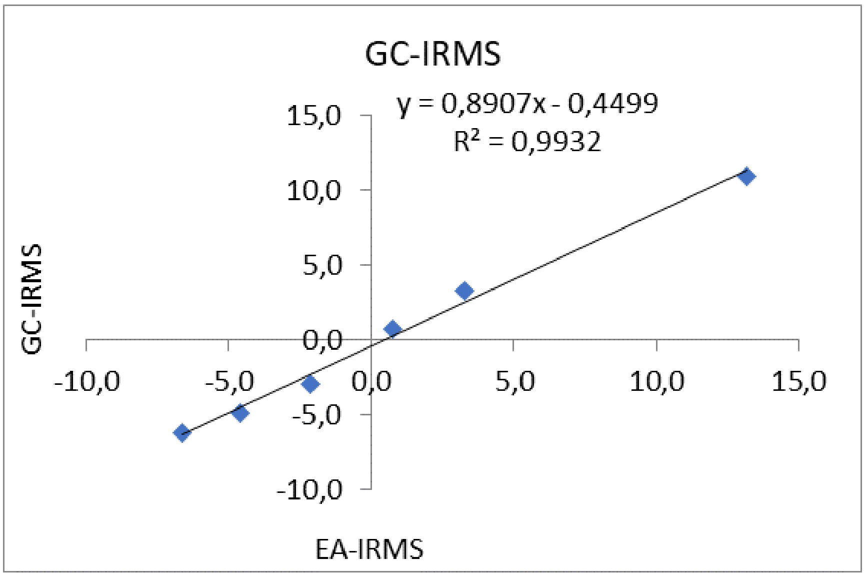

Figure S1: Example of standard calibration for AA corrections using EA-IRMS values vs GC-IRMS values of the STD.

Results: supplementary figures

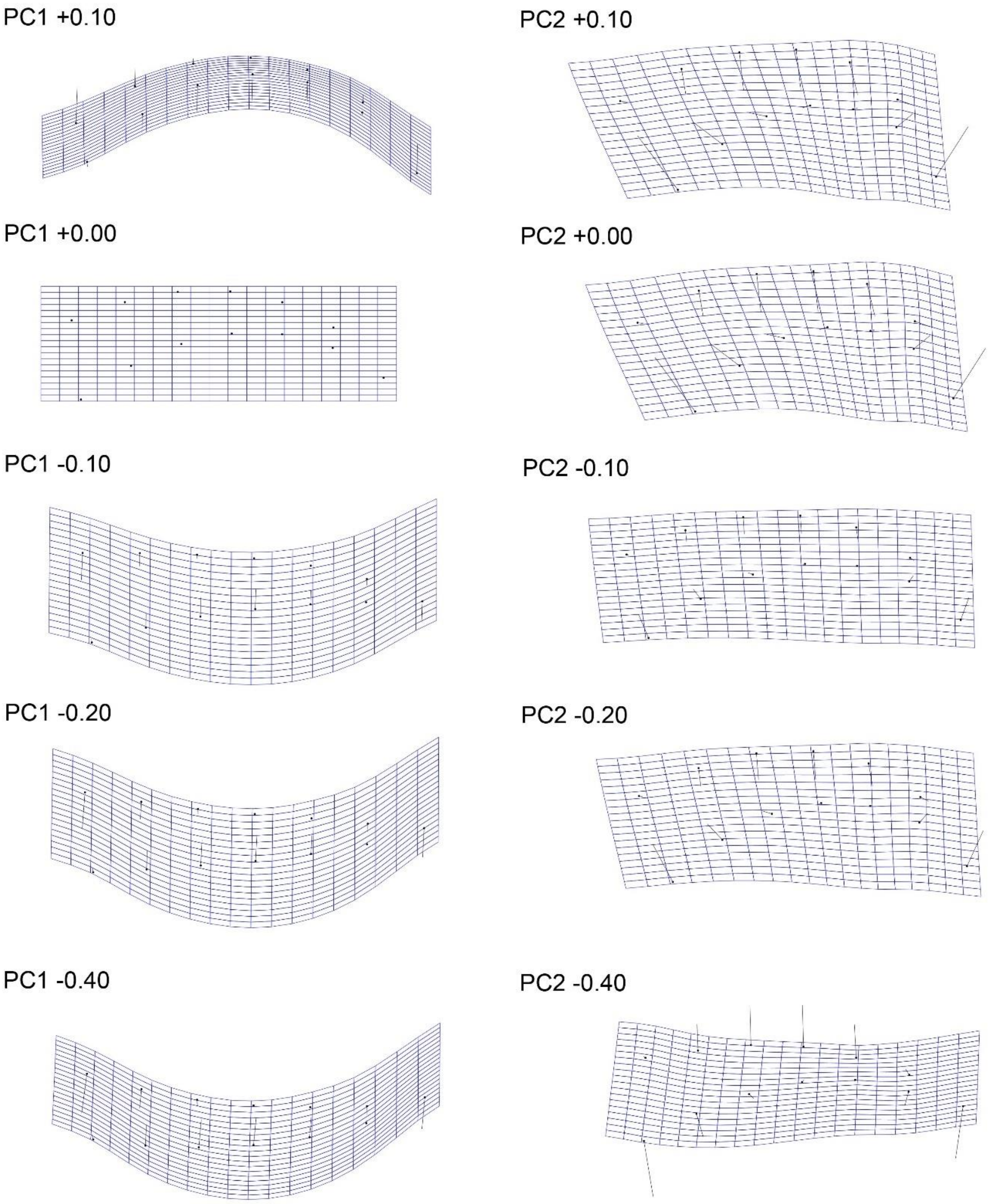

Figure S2: Thin-plate splines obtained by varying the PC1 and PC2, resulting in changes in bill curvature (PC1) or height/length (PC2).

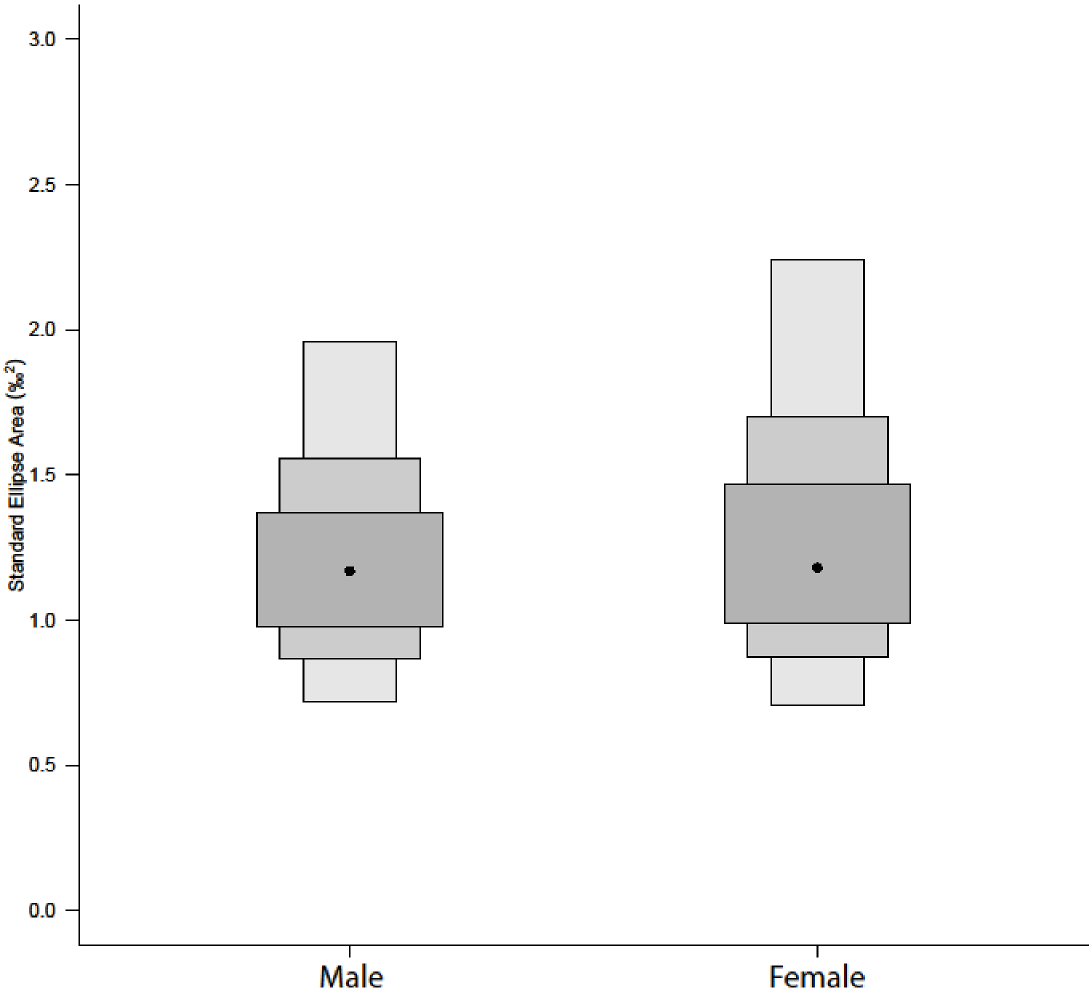

Figure S3: Density plots with 95% credible intervals comparing the width of isotopic niches of female and male huia, applying the SIBER package (Jackson et al, 2011).

Results: supplementary tables

Table S1: Results of the simple regression analyses testing the effect of sex on the principal components 1 and 2 obtained in the morphometric analyses.

**PC1**	**Estimate**	**s.e.**	**ndf**	**ddf**	***F*-value**	***p*-value**
Sex			1.00	82.00	507.73	< **0.01**
Female	0.07	0.004				
Male	-0.07	0.004				
**PC2**	**Estimate**	**s.e.**	**ndf**	**ddf**	***F*-value**	***p*-value**
Sex			1.00	82.00	0.09	0.76
Female	0.0004	0.003				
Male	-0.001	0.003				

Table S2: Results of the multiple regression analyses testing the effect of the principal components 1 and 2 on feather $\delta^{15}N_{bulk}$ and $\delta^{13}C_{bulk}$ values. Statistics are given for the point of exclusion of the term from the model.

**Feather $\delta^{15}N_{bulk}$**	**Estimate**	**s.e.**	**ndf**	**ddf**	***F*-value**	***p*-value**
PC1 : PC2	-1.37	112.40	1.00	26.00	0.0001	0.99
PC1	-3.49	2.08	1.00	27.00	2.82	0.10
PC2	17.43	8.864	1.00	28.00	3.87	0.06
**Feather $\delta^{13}C_{bulk}$**	**Estimate**	**s.e.**	**ndf**	**ddf**	***F*-value**	***p*-value**
PC1 : PC2	135.87	66.87	1.00	26.00	2.08	0.11
PC1	-3.05	1.28	1.00	28.00	6.69	0.02
PC2	-3.72	6.403	1.00	27.00	0.97	0.33

Table S3: Results of the simple regression analyses testing the effect of sex on feather $\delta^{15}N_{bulk}$ and $\delta^{13}C_{bulk}$ values.

**Feather $\delta^{15}N_{bulk}$**	**Estimate**	**s.e.**	**ndf**	**ddf**	***F*-value**	***p*-value**
Sex			1.00	28.00	4.85	**0.04**
Female	5.02	0.223				
Male	5.67	0.195				
**Feather $\delta^{13}C_{bulk}$**	**Estimate**	**s.e.**	**ndf**	**ddf**	***F*-value**	***p*-value**
Sex			1.00	28.00	6.00	**0.02**
Female	-22.77	0.14				
Male	-22.31	0.12				
**$\delta^{15}N_{bulk}$ and $\delta^{13}C_{bulk}$ (MANOVA)**	**Residuals**	**Pillai**	**ndf**	**ddf**	**F**	***p*-value**
Sex	28.00	0.28	2.00	27.00	5.27	**0.01**

Table S4: Results of the paired analyses testing the effect of sex on feather $\delta^{15}N_{bulk}$, $\delta^{13}C_{bulk}$ and both $\delta^{15}N_{bulk}$ and $\delta^{13}C_{bulk}$ values.

**Feather $\delta^{15}N_{bulk}$ (Wilcoxon paired test)**	**V**	***p*-value**

Sex	16.00			0.31
**Feather $\delta^{13}C_{bulk}$ (Wilcoxon paired test)**	**V**			***p*-value**
Sex	21.00			**0.03**
**$\delta^{15}N_{bulk}$ and $\delta^{13}C_{bulk}$ (Multivariate Hotelling one-sample test)**	**T2**	**df1**	**df2**	***p*-value**
Sex	20413.23	2.00	4.00	**<0.01**

Table S5: Results of the simple regression analysis and paired analysis testing the effect of sex on the birds' trophic position.

**TP**	**Estimate**	**s.e.**	**ndf**	**ddf**	***F*-value**	***p*-value**
Sex			1.00	10.00	0.10	0.75
Female	2.73	0.103				
Male	2.77	0.103				
**Feather $\delta^{15}N_{bulk}$ (Wilcoxon paired test)**					**V**	***p*-value**
Sex					10.00	1.00

Table S6: Results of the simple regression analysis testing the effect of sex on feather $\delta^{15}N_{AA}$ values. Amino acids: alanine (Ala), glycine (Gly), threonine (Thr), serine (Ser), valine (Val), leucine (Leu), isoleucine (Ile) proline (Pro), aspartic acid (Asx), glutamic acid (Glx), phenylalanine (Phe).

**$\delta^{15}N_{Ala}$**	**Estimate**	**s.e.**	**ndf**	**ddf**	***F*-value**	***p*-value**
Sex			1.00	2.00	7.95	0.11
Female	8.86	0.44				
Male	7.11	0.44				
**$\delta^{15}N_{Gly}$**	**Estimate**	**s.e.**	**ndf**	**ddf**	***F*-value**	***p*-value**
Sex			1.00	6.00	0.03	0.87
Female	3.51	0.69				
Male	3.37	0.53				
**$\delta^{15}N_{Thr}$**	**Estimate**	**s.e.**	**ndf**	**ddf**	***F*-value**	***p*-value**
Sex			1.00	7.00	1.59	0.25
Female	-21.02	2.85				
Male	-16.19	2.55				
**$\delta^{15}N_{Ser}$**	**Estimate**	**s.e.**	**ndf**	**ddf**	***F*-value**	***p*-value**
Sex			1.00	7.00	0.06	0.81
Female	-1.55	3.89				
Male	-2.88	3.48				
**$\delta^{15}N_{Val}$**	**Estimate**	**s.e.**	**ndf**	**ddf**	***F*-value**	***p*-value**
Sex			1.00	7.00	1.41	0.27
Female	8.24	3.26				
Male	13.43	2.92				
**$\delta^{15}N_{Leu}$**	**Estimate**	**s.e.**	**ndf**	**ddf**	***F*-value**	***p*-value**
Sex			1.00	8.00	2.65	0.14
Female	5.73	0.63				
Male	7.06	0.52				

$\delta^{15}N_{Ile}$	Estimate	s.e.	ndf	ddf	*F*-value	*p*-value
Sex			1.00	7.00	0.99	0.35
Female	10.44	2.55				
Male	13.85	2.28				
**$\delta^{15}N_{Pro}$**	**Estimate**	**s.e.**	**ndf**	**ddf**	***F*-value**	***p*-value**
Sex			1.00	10.00	0.82	0.39
Female	12.92	1.11				
Male	14.35	1.11				
**$\delta^{15}N_{Asx}$**	**Estimate**	**s.e.**	**ndf**	**ddf**	***F*-value**	***p*-value**
Sex			1.00	10.00	0.01	0.92
Female	7.68	1.04				
Male	7.83	1.04				
**$\delta^{15}N_{Glx}$**	**Estimate**	**s.e.**	**ndf**	**ddf**	***F*-value**	***p*-value**
Sex			1.00	10.00	0.03	0.86
Female	10.77	1.00				
Male	10.51	1.00				
**$\delta^{15}N_{Phe}$**	**Estimate**	**s.e.**	**ndf**	**ddf**	***F*-value**	***p*-value**
Sex			1.00	10.00	0.50	0.50
Female	6.06	0.68				
Male	5.38	0.68				

Dataset

Supplementary Material 2 is an Excel spreadsheet containing all specimens used in this study with their museum identifiers, collection data, bulk isotope measurements, compound specific (CSIA) values, geometric morphometrics coordinates and principal components. The eight huia pairs used for compound specific stable isotope analysis of nitrogen in amino acids (CSSIA-N-AA) are also specified.

Museum acronyms: CM = Canterbury Museum (Christchurch, New Zealand); CMNH = Carnegie Museum of Natural History (Pittsburgh, USA); LUOMUS = Luonnontieteellinen museo (Helsinki, Finland); MNHN = Muséum national d'Histoire naturelle (Paris, France); NMNZ = Museum of New Zealand Te Papa Tongarewa (Wellington, New Zealand); NHMD = Statens Naturhistoriske Museum (Copenhagen, Denmark); NHMW = Naturhistorisches Museum Wien (Vienna, Austria); NMW = Amgueddfa Cymru, National Museum Wales (Cardiff, UK); NRS = Naturhistoriska riksmuseet (Stockholm, Sweden); SMAG = Southland Museum and Art Gallery (Invercargill, New Zealand).

Column name	Description
LabelLocality	Details of the locality written on the specimen label
Collector	Details of the collector written on the speciment label
OtherLabelInformation	Other information in the speciment label
normalised_d15N	feather δ15Nbulk
normalised_d13C	feather δ13Cbulk
CN_mass_ratio	ratio feather δ13Cbulk and δ15Nbulk
CarbonCorrect	feather δ13Cbulk with Suess correction
Sex	Sex of the bird
LM1x.1	Bill landmark 1x
LM1y.1	Bill landmark 1y
LM2x.1	Bill landmark 2x
LM2y.1	Bill landmark 2y
LM3x.1	Bill landmark 3x
LM3y.1	Bill landmark 3y
LM4x.1	Bill landmark 4x
LM4y.1	Bill landmark 4y
LM5x.1	Bill landmark 5x
LM5y.1	Bill landmark 5y
LM6x.1	Bill landmark 6x
LM6y.1	Bill landmark 6y
LM7x.1	Bill landmark 7x
LM7y.1	Bill landmark 7y
LM8x.1	Bill landmark 8x
LM8y.1	Bill landmark 8y
LM9x.1	Bill landmark 9x
LM9y.1	Bill landmark 9y
LM10x.1	Bill landmark 10x
LM10y.1	Bill landmark 10y
LM11x.1	Bill landmark 11x
LM11y.1	Bill landmark 11y
LM12x.1	Bill landmark 12x
LM12y.1	Bill landmark 12y
LM13x.1	Bill landmark 13x
LM13y.1	Bill landmark 13y
PC.1	Principal component 1 for bill shape
PC.2	Principal component 2 for bill shape
PC.3	Principal component 3 for bill shape
PC.4	Principal component 4 for bill shape
PC.5	Principal component 5 for bill shape

PC.6	Principal component 6 for bill shape
PC.7	Principal component 7 for bill shape
PC.8	Principal component 8 for bill shape
PC.9	Principal component 9 for bill shape
PC.10	Principal component 10 for bill shape
PC.11	Principal component 11 for bill shape
PC.12	Principal component 12 for bill shape
PC.13	Principal component 13 for bill shape
PC.14	Principal component 14 for bill shape
PC.15	Principal component 15 for bill shape
PC.16	Principal component 16 for bill shape
PC.17	Principal component 17 for bill shape
PC.18	Principal component 18 for bill shape
PC.19	Principal component 19 for bill shape
PC.20	Principal component 20 for bill shape
PC.21	Principal component 21 for bill shape
PC.22	Principal component 22 for bill shape
PC.23	Principal component 23 for bill shape
PC.24	Principal component 24 for bill shape
PC.25	Principal component 25 for bill shape
PC.26	Principal component 26 for bill shape
Pair	Pair number
GlxPhe	Relationship feather δ15NAA glutamic acid and phenylalanine
TP	Trophic position
Alanine	feather δ15NAA alanine
Glycine	feather δ15NAA glycine
Threonine	feather δ15NAA threonine
Serine	feather δ15NAA serine
Valine	feather δ15NAA valine
Leucine	feather δ15NAA leucine
Isoleucine	feather δ15NAA isoleucine
Proline	feather δ15NAA proline
AsparticAcid	feather δ15NAA aspartic acid
GlutamicAcid	feather δ15NAA glutamic acid
Phenylalanine	feather δ15NAA phenylalanine

Script

Supplementary Material 3 is an R script containing the analyses presented in this manuscript and the codes for plotting the figures. It uses the dataset of the Supplementary Material 2.
